# Photoluminescence-Based Bioassay With Cysteamine-Capped TiO_2_ Nanoparticles for the Selective Recognition of *N*-Acyl Homoserine Lactones

**DOI:** 10.3389/fbioe.2021.750933

**Published:** 2021-12-03

**Authors:** Sahana Vasudevan, Parthasarathy Srinivasan, Prasanna Neelakantan, John Bosco Balaguru Rayappan, Adline Princy Solomon

**Affiliations:** ^1^ Quorum Sensing Laboratory, Centre for Research in Infectious Diseases (CRID), School of Chemical and Biotechnology, SASTRA Deemed to be University, Thanjavur, India; ^2^ Nanosensors Laboratory, School of Electrical & Electronics Engineering, Centre for Nanotechnology & Advanced Biomaterials (CeNTAB), SASTRA Deemed University, Thanjavur, India; ^3^ Department of Electronics and Communication Engineering, Amrita School of Engineering, AMRITA Vishwa Vidyapeetham Chennai Campus, Chennai, India; ^4^ Faculty of Dentistry, The University of Hong Kong, Pokfulam, Hong Kong SAR, China

**Keywords:** TiO_2_ nanoparticles, quorum sensing, acyl homoserine lactone, *Pseudomonas aeruginosa*, photoluminescence

## Abstract

Currently available diagnostic procedures for infections are laborious and time-consuming, resulting in a substantial financial burden by increasing morbidity, increased costs of hospitalization, and mortality. Therefore, innovative approaches to design diagnostic biomarkers are imperative to assist in the rapid and sensitive diagnosis of microbial infections. Acyl homoserine lactones (AHLs) are ubiquitous bacterial signaling molecules that are found to be significantly upregulated in infected sites. In this pioneering work, we have developed a simple photoluminescence-based assay using cysteamine-capped titanium oxide (TiO_2_) nanoparticles for AHL detection. The PL intensity variation of the oxygen defect state of TiO_2_ was used for the biosensing measurements. The bioassays were validated using two well-studied AHL molecules (C4-HSL and 3-oxo-C12 HSL) of an important human pathogen, *Pseudomonas aeruginosa*. The developed system has a maximum relative response of 98%. Furthermore, the efficacy of the system in simulated host urine using an artificial urine medium showed a linear detection range of 10–160 nM. Also, we confirmed the relative response and specificity of the system in detecting AHLs produced by *P. aeruginosa* in a temporal manner.

## 1 Introduction

Quorum sensing (QS) is a well-synchronized communication process that exists among bacteria to mediate the infection process in the host niches (Azimi et al., 2020). Such communications are established either through the chemical (*N*-acyl homoserine lactones; AHLs) or peptide (auto-inducing peptides; AIPs) cues by the Gram-negative and Gram-positive bacteria, respectively (Rutherford and Bassler, 2012). The quorum of bacterial pathogens directly correlates with the signal concentration, reaching a threshold to attract more and more respondents of its own (intra-species) or from other species (inter-species) with its specific receptor. More specifically, this group behavior often pertains to the upregulation of virulence genes altering the host niches towards a favorable environment for its survival.

It is well known that AHLs are the key players for Gram-negative bacterial communication. AHLs structurally consist of a lactone ring with varying lengths of carbon tail that are species-specific. The selectivity and specificity among the different AHLs produced by the respective Gram-negative organisms in the polymicrobial environment are based on the carbon length variation (Papenfort and Bassler, 2016). A classic example of AHL-based communication is *Pseudomonas aeruginosa*, which is the predominant infectious agent of cystic fibrosis (Lee and Zhang, 2015) and is also a dominant pathogen found in chronic wound infections (Klockgether and Tümmler, 2017), urinary tract infections (Mittal et al., 2009), and bacteremia (Tuon et al., 2012). This bacterium produces two AHL signals, C4-HSL and 3-oxo-C12 HSL. C4-HSL is synthesized by the AHL synthase enzyme, RhlI, and identified by the cognate receptor, RhlR. Similarly, 3-oxo-C12 HSL is synthesized and determined by LasI and LasR, respectively (Papenfort and Bassler, 2016).

Despite the close associations between these signaling systems, the receptors are specific only to their respective signaling molecules (Schuster et al., 2003). This has multiple advantages in terms of the genetic economy and monitoring the bacterial population. In addition to the activation of its virulence mechanism, AHL molecules can activate the gene expression of bacteria, which cannot produce signaling molecules (Subramoni and Venturi, 2009). Thus, AHL molecules help in maintaining the symbiotic relationship among different species. The presence of such AHL molecules is often found in clinical samples such as cystic fibrosis sputum (Azimi et al., 2020) and oral cavity (Muras et al., 2020). This forms a solid basis for our hypothesis that AHL molecules can be exploited as biomarkers for diagnosing an infectious environment. Since AHL molecules are extracellular products, with proper detection systems, they can be detected in a non-invasive manner. Previous studies on AHL detection were dependent on the bacterial biosensor methods (Kawaguchi et al., 2008; Struss et al., 2010; Wu et al., 2021) and the use of physicochemical techniques, which are time-consuming and require high-end instruments for detection (Kumari et al., 2008; Mukherji et al., 2013; Chahande et al., 2021). In addition, bacterial-based biosensors require periodical activation for a stable response (Steindler and Venturi, 2007). Recently, the use of nanomaterials for AHL detection was explored in different platforms. To detect food-borne pathogens, Sun and his coworkers studied the use of quantum dots and its inherent luminescence properties to detect AHL with improved selectivity and limits of detection, tested in the bacterial growth media conditions (Habimana et al., 2018; Yang et al., 2020). Furthermore, the same group explored electrochemical AHL biosensors using metal oxide nanostructures, specifically iron oxide nanomaterials combined with molecular imprinting technology (Jiang et al., 2016). These studies have opened the possibility of integrating nanotechnology-based optical biosensing in AHL detection.

In this context, the current study explores metal oxide nanostructures for AHL detection. Metal oxide nanostructures offer an excellent biosensing platform, owing to optical properties, ease of functionalization and morphological modifications (Solanki et al., 2011; Jo and Shin, 2020). Among the metal oxides, ZnO and TiO_2_ nanoparticles are very well exploited for optical biosensing applications, mainly photoluminescence-based biosensing. Our previous report on functionalized ZnO nanoparticles for AHL sensing proved that metal oxide nanostructured interfaces are the perfect choice for AHL sensing (Vasudevan et al., 2020). It was proved that oxygen vacancies are a vital factor for the AHL detection, which is substantial in TiO_2_ having a wide bandgap (3.0–3.2 eV) (Haider et al., 2019; Kang et al., 2019). Additionally, the isoelectric point of TiO_2_ (5.8) (McNamee et al., 2005) is much lower than that of ZnO (10.3) (Marsalek, 2014), which might play a decisive role in the interaction with cysteamine (Ma et al., 2020) and, thus, sensitivity. Considering these two parameters, the current work investigates the TiO_2_ metal oxide matrix with cysteamine for AHL sensing. TiO_2_ is most opted for its optical transmittance and chemical stability in harsh environments. Previous studies have reported the use of TiO_2_ nanostructures for PL-based detection of leucosis (Viter et al., 2012), BSA and DNA (Ensafi et al., 2015). TiO_2_ is much explored for the development of PL-based immunosensor to detect rabbit IgG and antigen of *Salmonella* sp. (Viter et al., 2017). In this context, the present study focuses on capping of TiO_2_ nanoparticles with an FDA-approved linker molecule, cysteamine, to detect AHL molecules. In addition to its role in critical physiological processes (Wan et al., 2011), this simple aminothiol has been used as a linker molecule due to its reactive groups (Sharon, 2014; Tayebi et al., 2016; Tavakkoli Yaraki et al., 2017; Oliva et al., 2018). In particular, there are reports that have augmented cysteamine as a linker molecule between TiO_2_ and gold nanoparticles to enhance the adsorption of gold nanoparticles onto the TiO_2_ surface (Park, 2011).

With this as the rationale, the present study aims to cap TiO_2_ nanoparticles with cysteamine for the effective detection of AHL molecules. Two variants of the AHL molecule, C4-HSL (short) and 3- oxo-C12 HSL (long), which are typically synthesized by *P. aeruginosa,* are chosen for the present study. The biosensing system established its selective and specific nature in the presence of artificial urine media (AUM) and the presence of *P. aeruginosa*. This interdisciplinary work combines the aspects of nanotechnology and microbiology to provide a fast and accurate diagnostic platform for the infectious environment. The advantages such as non-invasive sample collection, no sample processing and immediate results, overcome the shortcomings of the conventional microbiological techniques. Through this study, for the first time, we have demonstrated the real-time sensing of AHL through PL studies with cysteamine-capped TiO_2_ nanoparticles bioassay.

## 2 Materials and Methods

### 2.1 Synthesis and Functionalization of TiO_2_ Nanoparticles

Nanocrystalline TiO_2_ was synthesized using the hydrothermal method by employing titanium (IV) isopropoxide and sodium hydroxide (NaOH) as starting materials (Venkataprasad et al., 2020). Titanium (IV) isopropoxide (1 M) was added to 25 ml of distilled water, and subsequently, 2 M NaOH was added dropwise. This solution was kept under constant stirring for 30 min. The final volume was adjusted to 40 ml using distilled water. The hydrothermal reaction was carried out by heating of the solution up to 240°C for 12 h. Then, the final precipitate was washed several times with distilled water and dried at 450°C for 3 h to obtain nanocrystalline TiO_2_ nanoparticles.

The functionalization/capping was carried out by suspending the TiO_2_ nanoparticles and the linker molecule, cysteamine, in ethanol. Three different molar ratios of TiO_2_-Cysteamine (TiO_2_-Cys) were considered—1:0.5, 1:1, and 0.5:1—and sonicated for 12 h to obtain a homogenous cysteamine functionalized nanoparticle solution as reported in our previous study (Vasudevan et al., 2020).

### 2.2 Material Characterization

The structural analysis of the synthesized and functionalized nanoparticles was studied using an x-ray diffractometer (XRD, D8 Focus, Bruker, Germany). Oxidation states and atomic compositions of the synthesized and functionalized nanoparticles were examined using an x-ray photoelectron spectrometer (Thermo Fisher Scientific Inc., K Alpha, USA). Morphological studies of the synthesized and functionalized nanoparticles were characterized using a field emission transmission electron microscope (FE-TEM, JEM 2100 F, JEOL, Japan). The surface defects of the TiO_2_ nanoparticles and functionalized nanoparticles were investigated using a PL spectrophotometer (FP-8200, JASCO, USA). Functional group analysis of the prepared and functionalized nanoparticle was studied using the Fourier transform infrared spectrometer (FT-IR, Alpha-T, Bruker, Germany).

### 2.3 Bio-Analyte, Bacterial Culture, and Media Preparation

C4-HSL (N-Butyryl-DL-homoserine lactone, ≥96.0%) and 3-Oxo-C12-HSL [N-(3-oxododecanoyl)-homoserine lactone, ≥96.0%] were obtained from Sigma Aldrich, USA. The stock solutions of 1 M were prepared in dimethyl sulfoxide (DMSO, Sigma Aldrich).

The bacterial strain *P. aeruginosa* (MCC 3101) was maintained as a glycerol stock at −80°C. The AUM was prepared by adding the components (Supplementary Table S1) in the sterile distilled water and filter sterilized (Brooks and Keevil, 1997). The pH of the media was adjusted using 1 M NaOH (HiMedia Laboratories, USA) and concentrated hydrochloric acid solution (SRL, India).

### 2.4 Photoluminescence Biosensing Measurements

The detailed description of the biosensing measurements were carried out as described previously (Vasudevan et al., 2020) using PL spectrophotometer (FP-8200, JASCO, USA). The following are the PL parameters used by maintaining the emission and excitation bandwidth of 5 nm throughout. TiO_2_ nanoparticles were excited at the wavelength of 320 nm, and the emission spectra were observed in the range of 350–550 nm. Other conditions like the detector’s response time of 1 s and recording speed of 500 nm min^−1^ were maintained throughout. The PL spectra of TiO_2_ nanoparticles, TiO_2_-Cys, and TiO_2_-Cys in the presence of two AHLs (C4 and 3-oxo-C12) were recorded to determine the response. The relative response of the biosensor was calculated as below:

$$RR\;=\;\frac{R_{A}-\;R_{B}}{R_{A}}\;\times 100\%$$ (1)

Where *RR* denotes the relative response, *R*_*A*_ is the peak intensity of TiO_2_-Cys in the presence of AHL, and *R*_*B*_ denotes the peak intensity of TiO_2_-Cys in the absence of AHL. The AHL concentrations were varied from 10 to 40 nM to establish the linear response between the relative response and AHL concentration. For the host simulated environment, AUM, the same protocol was used. AHL concentrations from 10 nM–1 µM were dispersed in AUM and validated for the specificity and relative response.

### 2.5 Real-Time Analysis of TiO_2_-Cys With *P. aeruginosa*

TiO_2_-Cys was tested to identify the HSL synthesized by the clinical isolate of (human kidney stone) *P. aeruginosa*, MCC3101. The initial inoculum of the *P. aeruginosa* was prepared in AUM with OD595 = 0.05 and incubated aerobically at 37°C. The temporal profile of the PL spectra was recorded every 30 min up to 300 min to evaluate the bacterial growth-dependent sensing of the TiO_2_-Cys biosensing system.

## 3 Results and Discussion

### 3.1 Material Characterization of TiO_2_ and TiO_2_-Cys Nanoparticles

**FIGURE 1:**
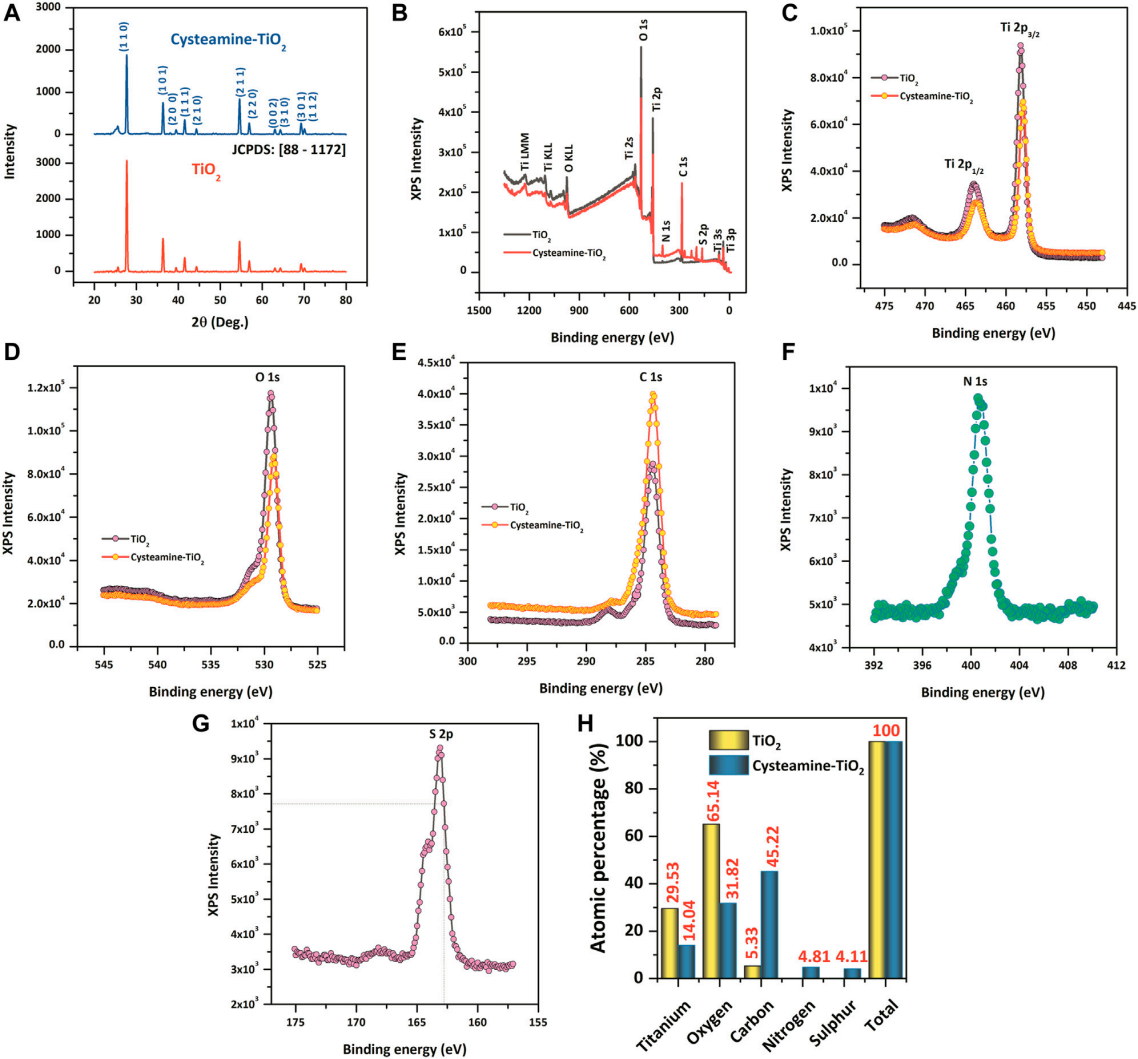
**(A)** XRD pattern of TiO_2_ and TiO_2_-Cys. **(B–G)** XPS spectrum of TiO_2_ and TiO_2_-Cys, Ti4+ oxidation state, O 1s, C 1s, N1s, and S 2p. **(H)** Comparative atomic percentages of each of the elements between TiO_2_ and TiO_2_-Cys.

Structural analysis of TiO_2_ and cysteamine-capped TiO_2_ nanoparticles are shown in Figure 1A. The observed XRD pattern was compared with the standard JCPDS card [88-1172], which revealed the formation of rutile phased TiO_2_ nanoparticles with tetragonal primitive lattice having lattice parameters of *a* = 4.566 Å and *c* = 2.948 Å [space group of P42/nm (136)]. The peaks at 2θ = 27.70°, 36.35°, 39.45°, 41.51°, 44.31°, 54.60°, 56.89°, 63.02°, 64.30°, 69.26°, and 70.06° correspond to (110), (101), (200), (111), (210), (211), (220), (002), (310), (301), and (112), respectively (Viana et al., 2010). The preferential plane orientation was found to be along the (110) plane for both TiO_2_ and cysteamine-capped TiO_2_ nanoparticles. However, cysteamine-capped TiO_2_ nanoparticles showed a lesser intense diffraction plane of (110) than TiO_2_, which, in turn, indicated the decreased crystallinity due to the active capping effect of cysteamine. The position of diffraction peaks corresponding to both TiO_2_ and cysteamine-capped TiO_2_ nanoparticles was shifted to the lower angles 2θ = ±0.2° due to the lattice mismatching, which, in turn, induce the lattice distortion.

Figure 1B displays the XPS survey spectra of TiO_2_ and cysteamine-capped TiO_2_ nanoparticles. The survey spectra of TiO_2_ nanoparticles confirmed the existence of elements such as Ti and O, which are consistent with the previous reports. The symmetric peak having the binding energies, 465.2 and 459.5 eV, attribute to Ti 2p_3/2_ and Ti 2p_1/2_, respectively (Hu et al., 2013; Park et al., 2015; Zhu et al., 2017). The binding energies and their difference in Ti 2p doublet of 5.8 eV confirmed the Ti^4+^ oxidation states (Diebold and Madey, 1996; Zhu et al., 2017). In addition, Figure 1D shows the peak of O 1s having a binding energy of 530.8 eV, which is in good agreement with the binding energy for TiO_2_ (Diebold and Madey, 1996; Mali et al., 2012). The capping of TiO_2_ by cysteamine was confirmed by the following: a reduction in the peak intensity of Ti (Figure 1C) and O (Figure 1D) for the TiO_2_-Cys, and an increase in the peak intensity of C 1s (1 (e)), N 1s (1(f)), and S 2p (1(g)) in the TiO_2_-Cys. It should be noted that there were no new peaks formed, which may be due to the absence of a covalent bond between cysteamine and TiO_2_ (Abbas et al., 2020). An increase in the atomic percentages of carbon (45.22%), nitrogen (4.81%), and sulfur (4.11%) was observed.

**FIGURE 2:**
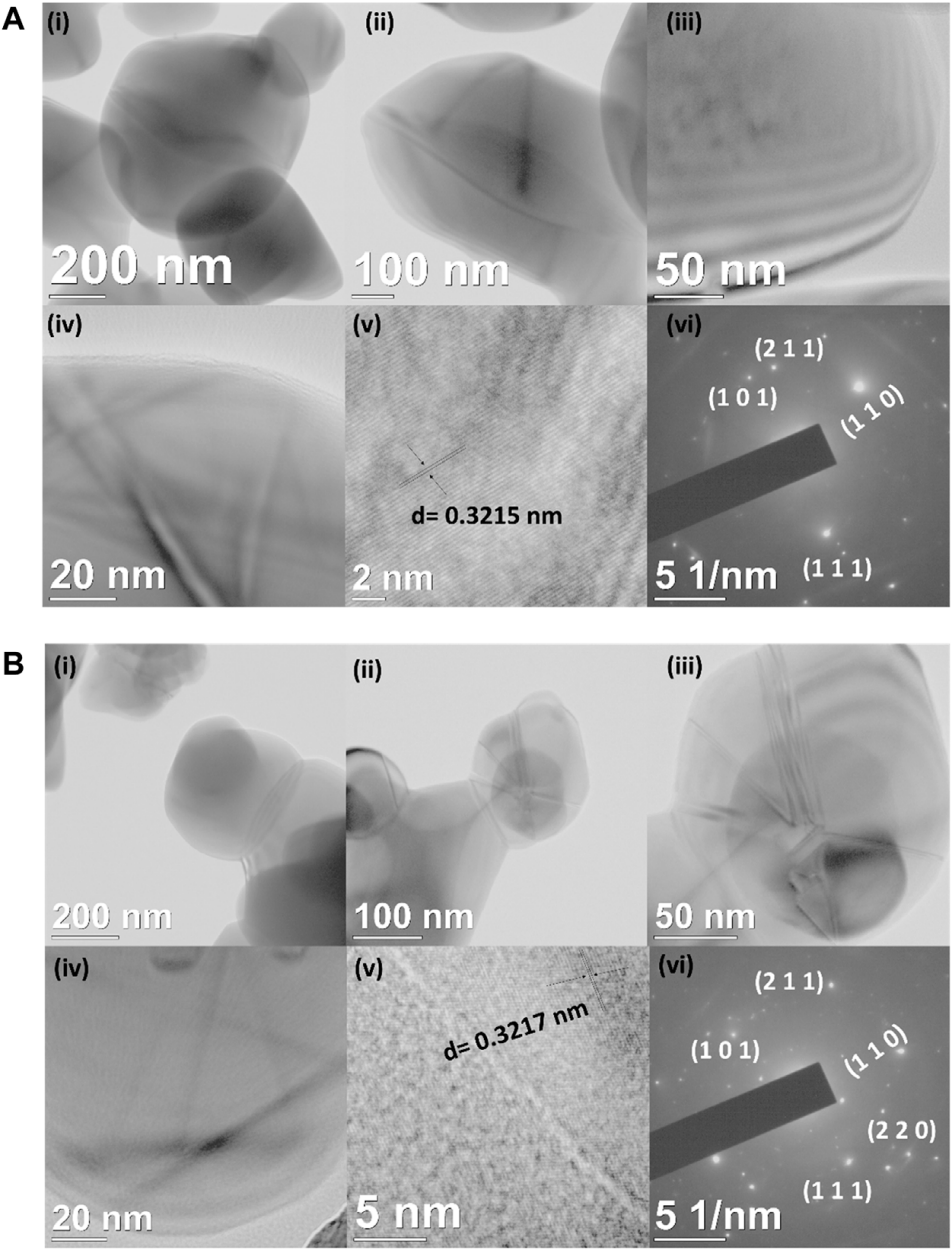
**(A) (i–iv)** HR–TEM micrographs of TiO_2_ nanoparticles at different magnifications. **(v)** Interplanar distance and **(vi)** SAED pattern of TiO_2_ nanoparticles. **(B) (i–iv)** HR–TEM micrographs of TiO_2_-Cys nanoparticles at different magnifications. **(v)** Interplanar distance and **(vi)** SAED pattern of TiO_2_-Cys nanoparticles.

Furthermore, the morphology, size, and lattice structure were observed by TEM analysis. Figures 2A,B shows the TEM micrographs of TiO_2_ nanoparticles and TiO_2_-Cys at different magnifications. Figure 2Ai–iv shows the formation of nanogranular morphology of the TiO_2_ nanoparticles. Figure 2Av shows the high-resolution TEM image representing the lattice fringe with the interplanar distance of *d* = 0.321 nm (Wang et al., 2013; Huyen et al., 2018), and the SAED (Selected Area Electron Diffraction) pattern in Figure 2Avi confirmed the polycrystalline nature of the synthesized TiO_2_ nanoparticles. The TEM results are consistent with the XRD data. The functionalization of cysteamine is a physical attribution process in the present case, and the cysteamine molecules are highly active enough over the surface of TiO_2_ nanograins. Similarly, the polycrystallinity was observed from the SAED pattern shown in Figure 2Bvi. Previous studies have shown the effect of calcination temperature and thus the effect of size on the optical properties of TiO_2_ nanoparticles (Lin et al., 2006; Pan et al., 2013; Horti et al., 2019) At higher calcination temperatures, the size of the nanoparticles and the induced oxygen vacancies are prominent (Horti et al., 2019). A similar result is observed, at the calcination temperature of 450°C, the average size of the TiO_2_ nanoparticles obtained was ∼115 nm (Supplementary Figure S1). The PL behavior is obtained similarly, and the oxygen vacancy (peak at 468 nm) is prominent.

### 3.2 Photoluminescence-Based Bioassay for AHL Detection

#### 3.2.1 Optimization of TiO_2_-Cys ratio for AHL Response

**FIGURE 3:**
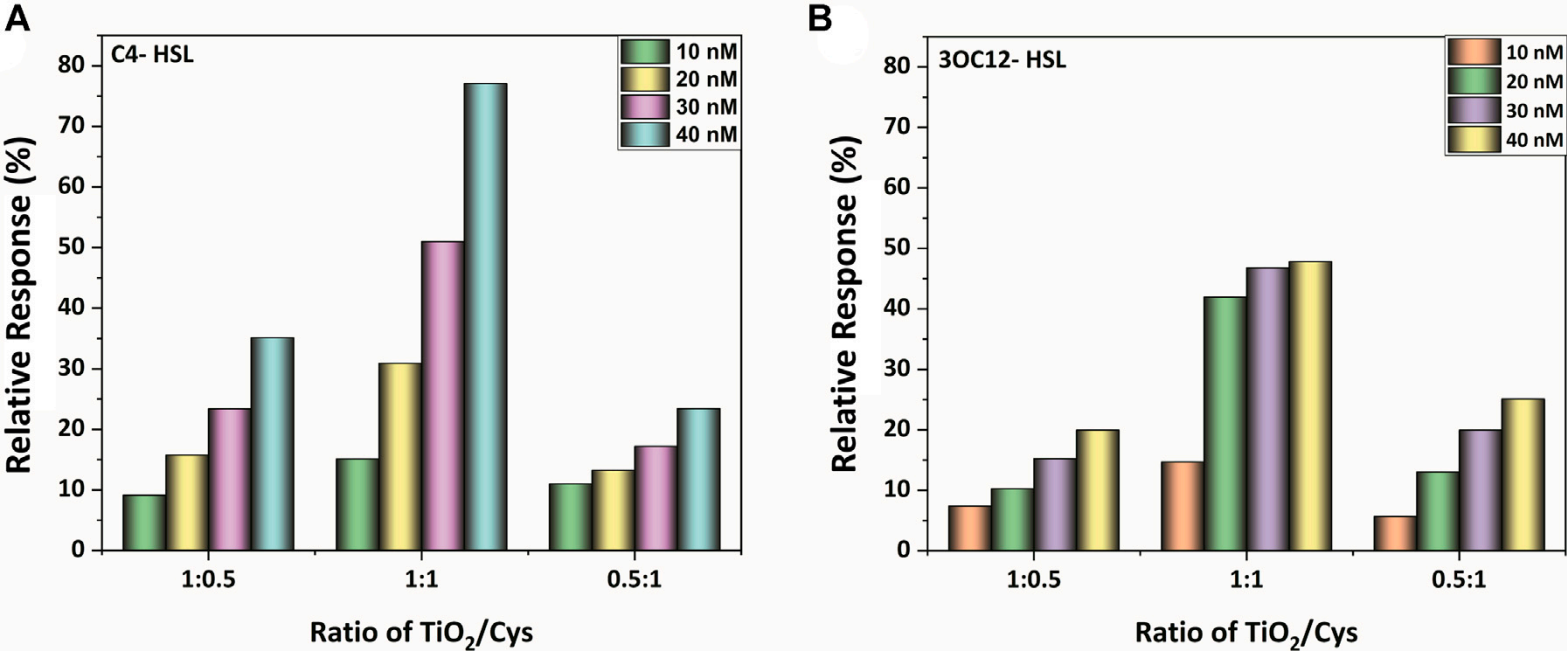
The relative response profile of the TiO_2_-Cys system at molar ratios of 1:0.5, 1:1, and 0.5:1, respectively, at varying concentrations of AHL molecules: **(A)** C4-HSL, **(B)** 3-oxo-C12-HSL.

From the structural characterization methods, capping of the cysteamine around TiO_2_ nanoparticles is evident. Furthermore, to evaluate the maximum biosensing outcome, the PL spectrum was observed for the three different molar ratios of TiO_2_-Cys (0.5:1, 1:1, and 1:0.5). Figures 3A,B show the relative response profile of the various ratios for the increasing concentrations (10–40 nM) of the bioanalyte, C4-HSL, and 3-Oxo-C12 HSL, respectively. The results demonstrated that 1:1 is the optimal ratio of the TiO_2_-Cys for both the AHLs considered. The relative response was dependent on the concentration of AHL molecules, with a maximum value of 80% obtained for C4-HSL. At this ratio, the optimal exposure of the defect centers to cysteamine was achieved, which significantly improved the relative response of the biosensing system. A similar trend was previously reported for ZnO-Cys (Tavakkoli Yaraki et al., 2017; Vasudevan et al., 2020). While the concentration of cysteamine was less, it was not sufficient to interact with the defect centers of the TiO_2_ nanoparticles and, in turn, was insufficient to detect the AHL molecules, leading to the decreased relative response. On the other hand, when the cysteamine concentration exceeds the TiO_2_ nanoparticles, the reduced relative response was due to the interaction of cysteamine with most of the defect centers. Hence, upon the addition of AHL molecules, the presence of excessive cysteamine led to a reduced relative response. Thus, for further studies, a 1:1 ratio of TiO_2_-Cys was considered.

#### 3.2.2 Spectral Profile of TiO_2_-Cys Towards AHL Detection

**FIGURE 4:**
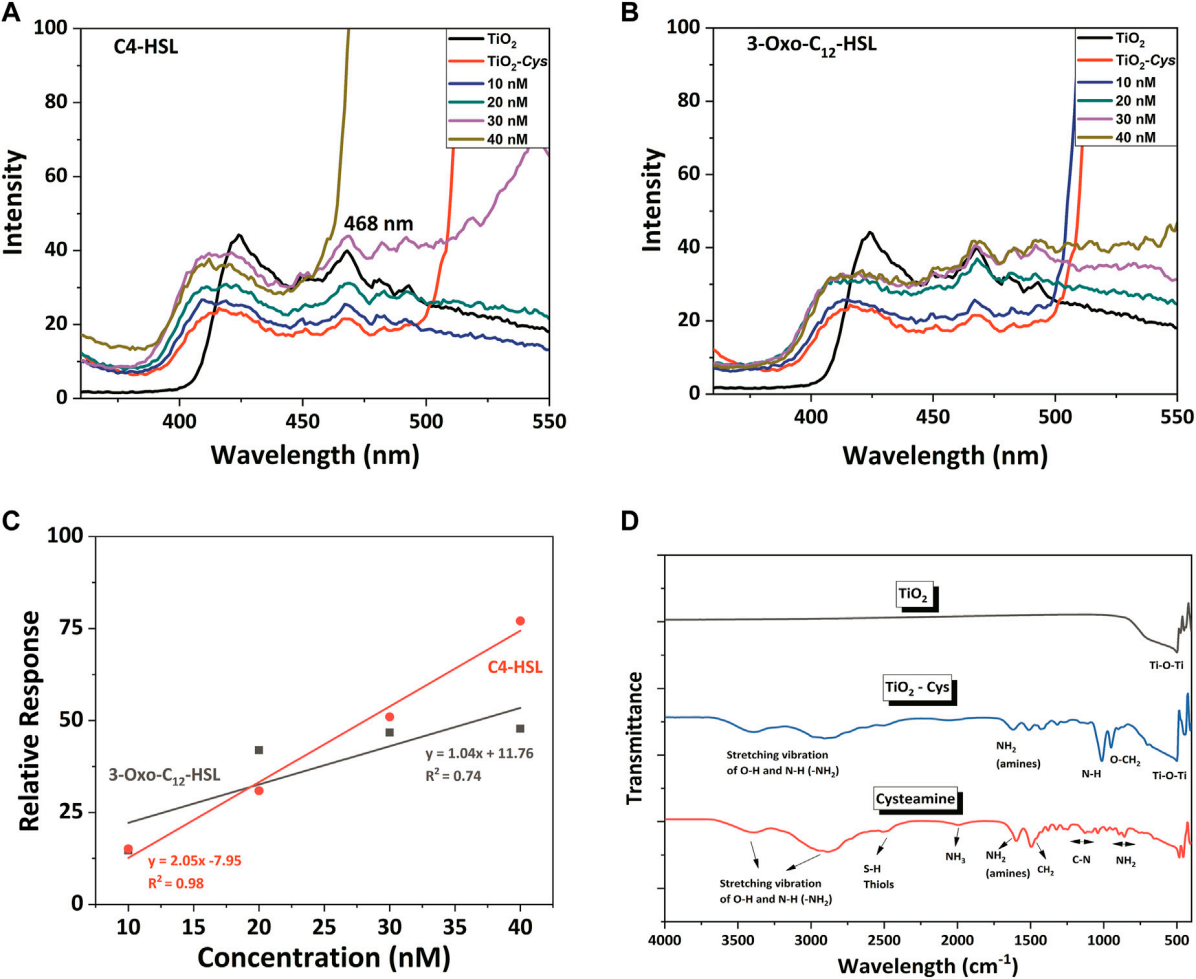
The PL spectral profile of TiO_2_, TiO_2_–Cys system with AHLs: **(A)** C4-HSL and **(B)** 3-oxo-C12-HSL. **(C)** Linearity fitting between relative response and AHLs concentration. **(D)** FTIR spectrum of TiO_2_, TiO_2_-Cys and Cysteamine.

Figure 4 depicts the PL spectral defect profile of the synthesized TiO_2_ nanoparticles excited at the wavelength of 320 nm. It is known that the PL behavior is different for the anatase and the rutile phase (Pallotti et al., 2017). The XRD analysis of the current study reported that the TiO_2_ nanoparticles is in its rutile phase. Even though the exact mechanism of the rutile PL spectrum is poorly understood, several researchers explain two hypotheses: free holes mechanism (Knorr et al., 2008; Rex et al., 2016) and trapped holes (Nakamura and Nakato, 2004; Nakamura et al., 2005) mechanism. A recent report decoded the rutile PL mechanism and claimed that recombination of free carriers with the valence band holes initiates the rutile PL where the enhancement of PL is caused by the O_2_ adsorption (Pallotti et al., 2017).

Considering the current study, the following intrinsic defect states were obtained as excitation peaks in the PL spectrum: 424, 468, 484, and 493 nm. The peak at 424 nm is due to the self-trapped excitons (STE) of the TiO_6_^2-^ octahedron and the recombination of free excitons. This is generated by the lattice localized electron upon the excitation at 320 nm, which captures a hole (Rathore et al., 2021). The emission peak at 468 nm, equivalent to 2.65 eV, is attributed to the surface oxygen vacancies and defects (Akshay et al., 2019b; Rathore et al., 2021). While the peak at 484 nm is due to the blue-green emission band that is attributed to the de-excitation to acceptor surface defects (Prajapati et al., 2017; Akshay et al., 2019a), the peak at 491 nm is due to the charge transition associated with oxygen defects (Choudhury and Choudhury, 2014). Among the observed peaks, the peak at 468 nm of surface oxygen vacancies and defects accounted for the AHL detection, due to its prominence over the other peaks. Figures 4A,B depict the PL spectrum recorded for the TiO_2_, Cysteamine-capped TiO_2_ and TiO_2_-Cys in the presence of an increasing concentration of the AHL molecules. The studies tested the detection of the AHL molecules: C4-HSL and 3-Oxo-C12 HSL molecules, which are indigenously produced by the pathogen *P. aeruginosa.* It should be noted that the capping of TiO_2_ nanoparticles by cysteamine has significantly quenched the luminescence of TiO_2,_ possibly masking the oxygen defect centers. However, upon the addition of AHL, there was a concentration-dependent increase in the intensity of peak at 468 nm observed in the spectral profile. As previously proven for the ZnO-Cys system (Vasudevan et al., 2020), the carbonyl group of AHL and the amine group of the cysteamine possibly interact. This exposes the defect centers of TiO_2_, thereby increasing the PL emission intensity by adding the AHL molecules. To ensure that free cysteamine does not interfere with the sensing process, PL was recorded at the same conditions at different concentrations of C4-HSL (Supplementary Figure S2). It was observed that there was no peak at 468 nm detected, in the given excitation wavelength. This confirms that no interfering effect is observed in the response of the developed bioassay because of the free ligands.

Among the two AHLs considered, C4 -HSL showed a higher relative response than 3-Oxo-C12. The only structural difference between the two AHLs is the carbon chain length and associated hydrophobicity. Thus, the PL intensity is shown to be sensitive to the hydrophobicity of the AHL molecules. The varying concentrations of AHL molecules from 10 to 40 nM were taken for the sensing studies. In general, adding AHL molecules increased the intensity of the 468-nm peak in a concentration-dependent manner. In particular, the C4-HSL exhibited increased intensity at the concentration greater than 30 nM compared to TiO_2_ and TiO_2_-Cys. However, the 3-Oxo–C12 HSL showed a marginal increase of PL intensity at a concentration above 30 nM that of the TiO_2_ but more than that of TiO_2_-Cys. A similar trend was observed for the ZnO-Cys, which proves the role of the AHL carbon chain in determining the interaction between the amine group of cysteamine and AHL molecules. The linear relationship between the relative response and the AHL concentration was linearly fit in the following equations:

$$\text{C}4-\text{HSL:}y=2.05x-7.95\;,R^{2}=0.98$$ (2)

$$3‐\text{oxo}‐{\text{C}}_{12}\text{HSL}:y=1.04x\;+11.76,\;R^{2}=0.74$$ (3)

In the case of C4-HSL, the *R* ^2^ approaching 0.98 very well establishes the linear relationship between the relative response and the AHL concentration. In the previous study with ZnO-Cys, the linearity of the biosensing system was proven for all the AHL molecules considered, despite reduced relative response in the case of long-chain AHL molecules. A similar result was obtained for the current case with TiO_2_-Cys where it is highly linear for short-chain AHL molecules and comparatively less linear in the case of long-chain AHL molecule considered.

**FIGURE 5:**
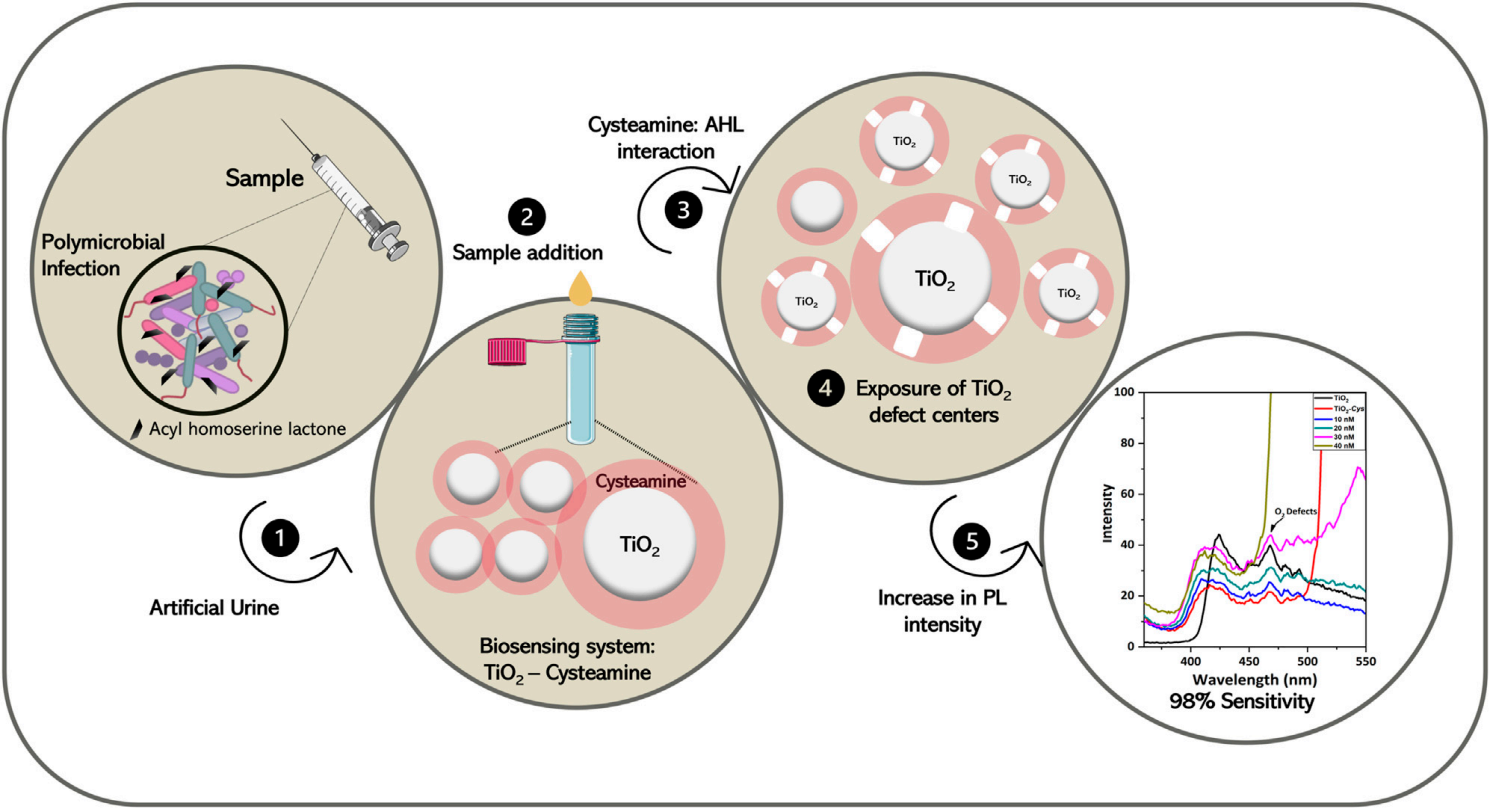
Graphical representation of the overall workflow.

The interaction of TiO_2_ and cysteamine was decoded by observing the FTIR spectrum (Figure 4D). The O-Ti-O functional group was proved from the characteristic peak at 503 cm^−1^ for TiO_2_ and TiO_2_-Cys (Erdem et al., 2001; Padmanabhan et al., 2007; Zhang et al., 2018). The presence of cysteamine was confirmed by thiol and amines (NH_2_), having peaks at 2,499 and 1,600 cm^−1^, respectively (Vasudevan et al., 2020). As observed in our previous report, the peak corresponding to the S-H thiol group was absent in the case of the TiO_2_-Cys system, which confirmed the capping effect of TiO_2_ by cysteamine through thiol linkages. This also supported the optimal ratio of 1:1 of TiO_2_-Cys. Another point to be noted is the presence of the NH_2_ group in the TiO_2_-Cys, which is proven to have strong interaction with the carbonyl group. Thus, like the ZnO-Cys system, the TiO_2_-Cys system interacts with each other through the thiol group, exposing the amine functional group. This possibly facilitates the interaction of the amine group with the carbonyl chain of the AHL molecule leading to the enhanced PL intensity at 468 nm. It should be noted that the presence of the amine functional group is not prominent as observed previously for the ZnO-Cys system. This is possibly due to the dominance of electrostatic interactions between TiO_2_ and cysteamine as reported previously (Park, 2011). TiO_2_ has an isoelectric point of 5.8 (McNamee et al., 2005) which has a strong electrostatic interaction with cysteamine having an isoelectric point of 9.5 (Ma et al., 2020). Thus, it is evident that the isoelectric points play a role in the enhanced electrostatic interaction between cysteamine and TiO_2_. Thus, the combination of thiol linkage and electrostatic interactions facilitates the stable capping of cysteamine. Figure 5 depicts the graphical representation of the biosensing mechanism.

#### 3.2.3 Spectral Profile of TiO_2_-Cys Towards AHL Detection in Simulated Host Environment: Artificial Urine Media

The relative response and the selectivity of TiO_2_-Cys were further evaluated in the presence of a simulated host environment—AUM. AUM was prepared (Supplementary Table S1) as previously reported by Brooks and Keevil (1997). This study evaluates the selectivity of the sensing system in the presence of possible interference molecules. pH is the important factor that differentiates the healthy and the diseased condition (Lai et al., 2019). It was shown that for complicated urinary tract infections, pH moves towards the alkaline zone, whereas for uncomplicated UTI, the pH is almost maintained neutral (Yaxley, 2016). The reported composition of AUM was stable at pH 6.5, and upon an increase in pH, salts precipitate, leading to an unstable condition (Brooks and Keevil, 1997). However, to account for the relative response of the developed biosensing system concerning the varying pH, AUM was prepared at different pH—6.5, 7, and 8. The relative response profile was measured at a concentration of 160 nM. This concentration was chosen since C4-HSL and 3-oxo-C12 HSL exhibited maximum relative response at 160 nM in the AUM medium (pH 6.5).

**FIGURE 6:**
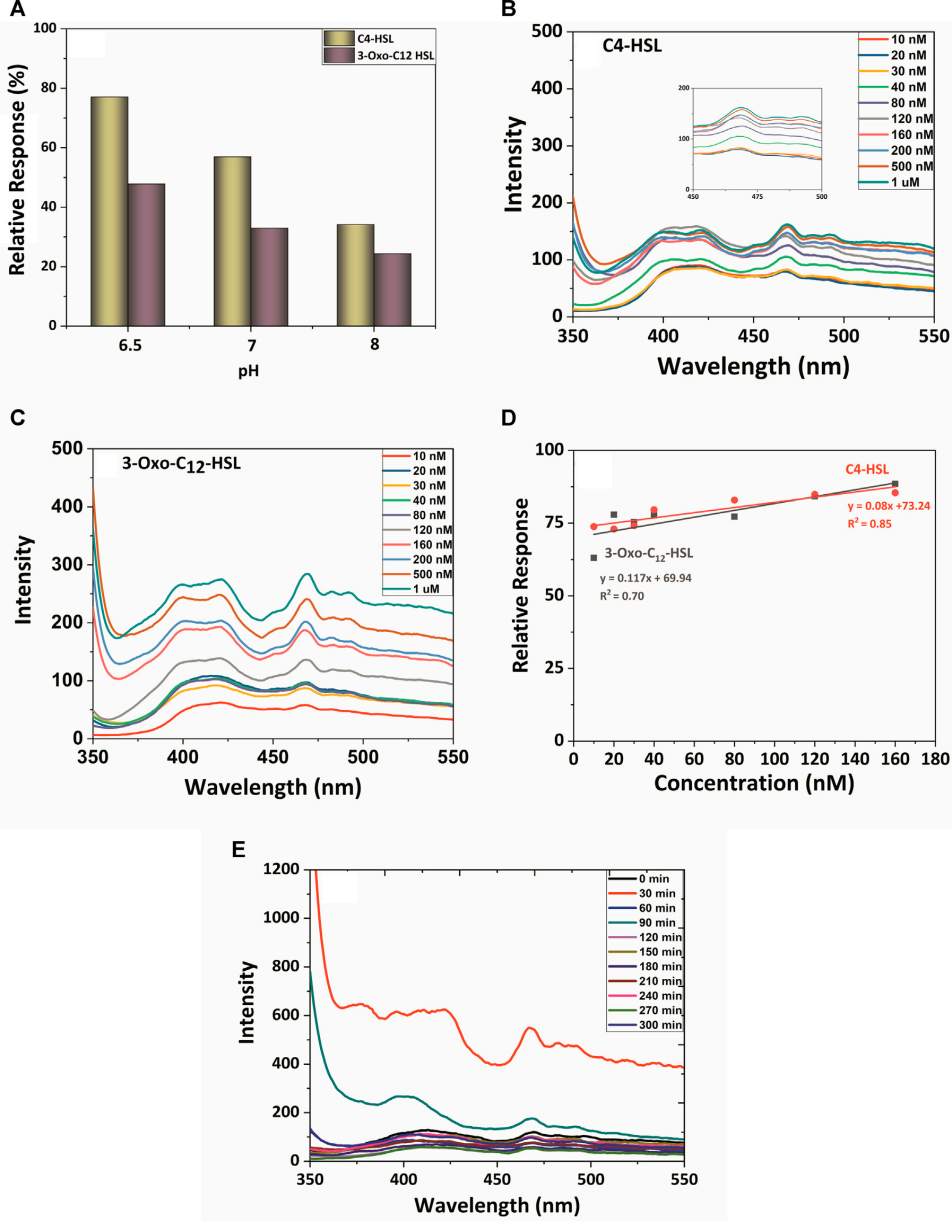
**(A)** pH role on relative response profile: The PL spectral profile of TiO_2_, TiO_2_-Cys system with AHLs in AUM: **(B)** C4-HSL and **(C)** 3-oxo-C12-HSL. **(D)** Linearity curve between relative response and AHLs concentration. **(E)** The PL spectral and temporal profile of the TiO_2_-Cys system with *P. aeruginosa* (MCC3101).

Figure 6A shows that the biosensing system TiO_2_-Cys provided a stable response in the simulated host environment at pH 6.5. As expected, the maximum relative response for C4-HSL and 3-Oxo-C12 HSL was obtained at AUM having pH 6.5. There was a decreasing trend in the relative response of the biosensing system as the pH increased. The precipitation of salts at the increased pH is possible for this trend (Brooks and Keevil, 1997). Also, the previous study proved that the electrostatic interaction between TiO_2_ and cysteamine is dependent on the pH largely (Park, 2011). Thus, in addition to the AUM stability at pH 6.5, the electrostatic interactions between TiO_2_ and cysteamine contributed to the enhanced interaction with the AHL molecule at the pH of 6.5. The relative response of the biosensing system in varying pH needs further studies on the actual urine samples.

At the stable pH 6.5 of AUM, the PL spectrum profile was measured for the TiO_2_-Cys biosensing system (Figures 6B,C). This study also accounted for the performance of the biosensing system in the presence of possible interferences in the host environment, including salts, metals, and other components. The spectrum profile highlighted the signature peak of 468 nm in AUM. In the AUM environment, carbon-chain length and associated hydrophobicity played an important role in the specificity of the system (Figure 6D). The response recorded for C4-HSL was more profound when compared to the long-chain AHL, 3-Oxo–C12 HSL. Upon increasing concentrations from 10 nM to 1 μM, there was an increase in PL intensity. A closer look in the relative response by fitting the linear response (Eqs 4, 5) between the concentration and relative response revealed that the biosensing system attained maximum relative response at 160 nM, which led to the saturation in the response:

$$C4-\text{HSL}:y=0.08x+73.24\;,\;R^{2\;}=0.85$$ (4)

$$3‐\text{oxo}‐{\text{C}}_{12}\text{HSL}:y=0.117x\;+69.94,\;R^{2}=0.70$$ (5)

The reduction in the linearity (*R* ^2^) is possibly due to the stability of AUM at pH 6.5. Even then, linear response with *R* ^2^ values 0.85 was observed for C4-HSL, and a slight reduction in 3-oxo–C12 was observed having an *R* ^2^ value of 0.7.

#### 3.2.4 Real-Time Biosensing Profile

The robustness of the biosensing system was validated by measuring the PL response with *P. aeruginosa,* which naturally produces two AHL molecules: C4-HSL and 3-Oxo- C12 HSL in a time-dependent manner. It should also be noted that the *Pseudomonas* sp. is known to produce several secondary metabolites, besides the AHL molecules (Gross and Loper, 2009). The real-time profiling also validates the selectivity of the sensing system in the presence of these secondary metabolites and the urine components.

*P. aeruginosa* (MCC 3101), a model micro-organism, was inoculated in AUM, and the response of the biosensing system was measured for every 30 min till 5 h (Figure 6E). The release and accumulation of AHL molecules are density-dependent; i.e., the increase in the number of cells increases the AHL molecules accumulation (Papenfort and Bassler, 2016). The TiO_2_-Cys system sensed the AHL molecules released by the bacteria with the maximum intensity of 468 nm peak at the time point of 30 min. While a time-dependent linear response was foreseen, the peak intensity was decreased after 30 min, itself. This was due to the inherently dynamic nature of the biological system. The released AHL molecules are primarily and most specifically detected by the bacterial quorum sensing receptors to activate QS (Papenfort and Bassler, 2016). This leaves the undetected molecules available for our biosensing system to detect. Hence, there was a possible decrease in the peak intensity in the later time points. However, this might not be the case in real conditions. The polymicrobial environment having bacterial species that produced other different AHL molecules will also be present along with the considered AHL molecules. Thus, further studies on different AHL-producing strains and in different host-simulated environments are being carried out to elaborate the specificity and sensitivity of the TiO_2_-Cys biosensing system to detect AHL molecules and thereby diagnose infections effectively.

## 4 Conclusion

The alarming increase in life-threatening infections necessitates the growing need for immediate infection diagnosis. This requires the advent of point-of-care diagnostics that are rapid and non-invasive with minimal or no sample processing. In this regard, this is the first study to report the bioassay based on cysteamine-capped TiO_2_ nanoparticles to identify the quorum sensing signaling molecule, AHL. The characterization analysis revealed the capping of cysteamine around TiO_2_ nanoparticles. This capping by cysteamine quenches the TiO_2_ photoluminescence. The external addition of AHL unmasks the oxygen defect states and thereby enhancing the PL intensity at 468 nm. The highlighting aspect of the study is that the selectivity and the specificity of the bioassay were proven in the host simulated condition—AUM. Through this study, the role of oxygen vacancies is well established in AHL sensing. The host sensing environment and difference in the isoelectric point determine the choice of metal oxide nanostructures and the linker molecule. The lesser the difference between isoelectric points, the more the relative response for AHL detection. Further studies are in progress to explore metal oxides, different linker molecules with varying isoelectric points, electronic bandgaps, and strong evidence of interaction between cysteamine and interface materials.

## Data Availability

The original contributions presented in the study are included in the article/Supplementary Material. Further inquiries can be directed to the corresponding authors.
