# Supplementary material for: Photoluminescence-Based Bioassay With Cysteamine-Capped TiO2 Nanoparticles for the Selective Recognition of N-Acyl Homoserine Lactones
Source: Front Bioeng Biotechnol. 2021 Dec 3;9:750933. doi: 10.3389/fbioe.2021.750933 (PMC8678280; doi:10.3389/fbioe.2021.750933)
Supplement: Supplementary file 1 [file DataSheet1.DOCX]

**Photoluminescence-based bioassay with Cysteamine Encapsulated TiO_2_ Core-Shell for the Selective Recognition of N- Acyl Homoserine Lactones**

Sahana Vasudevan^1^, Parthasarathy Srinivasan^2^, Prasanna Neelakantan^3^, John Bosco Balaguru Rayappan*^2^, Adline princy Solomon*^1^

---------------------------------------------------------------------------------------------------------------------

**Table S1** Components of Artificial Urine Media^1^

| **S.No.** | **Component** | **Quantity (g/L)** |
| --- | --- | --- |
| 1. | Peptone | 1 |
| 2. | Yeast Extract | 0.005 |
| 3. | Lactic Acid | 0.1 |
| 4. | Citric Acid | 0.4 |
| 5. | Sodium Bicarbonate | 2.1 |
| 6. | Urea | 10 |
| 7. | Uric Acid | 0.07 |
| 8. | Creatinine | 0.8 |
| 9. | Calcium Chloride.2H_2_O | 0.37 |
| 10. | Sodium chloride | 5.2 |
| 11. | Iron II sulphate.7H_2_O | 0.0012 |
| 12. | Magnesium Sulphate.7H_2_O | 0.49 |
| 13. | Sodium Sulphate.10H_2_O | 1.6 |
| 14. | Potassium-Di-hydrogen Phosphate | 0.95 |
| 15. | Di-potassium hydrogen phosphate | 1.2 |
| 16. | Ammonium Chloride | 1.3 |

**References:**

1 T. Brooks and C. W. Keevil, *Lett. Appl. Microbiol.*, 1997, **24**, 203–206.


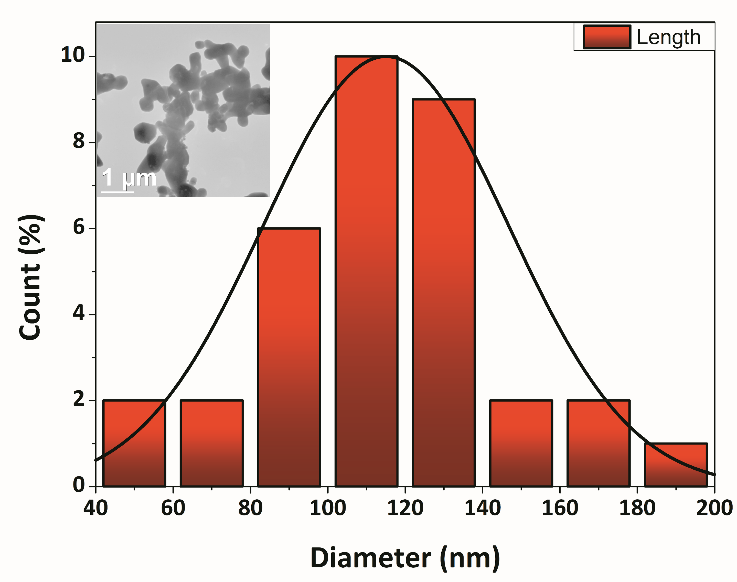


**Fig. S1**: Size Distribution Graph of the TiO_2_ nanoparticles. TEM image of the nanoparticles (inset)


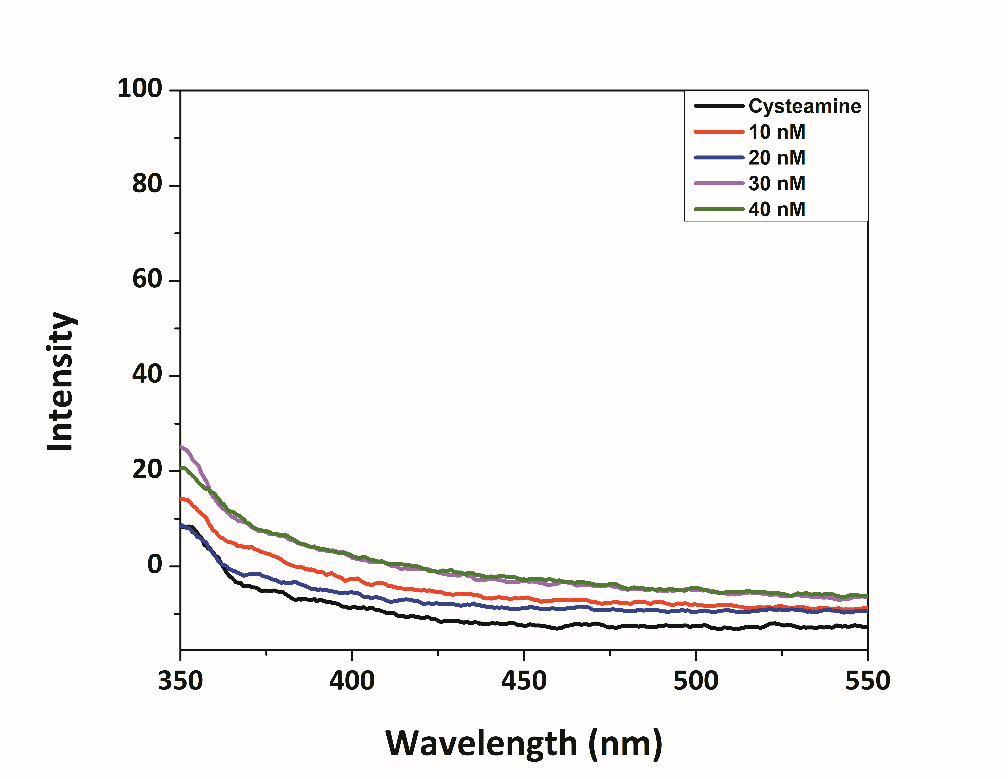


**Fig S2:** PL spectral profile of Cysteamine with C4-HSL.
